# Factors related to daily use of the paretic upper limb in patients with chronic hemiparetic stroke–A retrospective cross-sectional study

**DOI:** 10.1371/journal.pone.0247998

**Published:** 2021-03-09

**Authors:** Syoichi Tashiro, Miho Kuroki, Kohei Okuyama, Osamu Oshima, Miho Ogura, Nanako Hijikata, Takuya Nakamura, Asako Oka, Michiyuki Kawakami, Tetsuya Tsuji, Meigen Liu

**Affiliations:** 1 Department of Rehabilitation Medicine, Keio University School of Medicine, Shinjuku, Tokyo, Japan; 2 Department of Rehabilitation Medicine, Kyorin University School of Medicine, Mitaka, Tokyo, Japan; University of Tokyo, JAPAN

## Abstract

**Aims:**

The present study aimed to determine factors associated with the frequency of paralyzed upper extremity (UE) use in chronic stroke patients with severe UE functional deficiency.

**Methods:**

We retrospectively reviewed the medical records of 138 consecutive patients, and 117 was analyzed (median age, 55 [range, 18–85] years; median stroke duration, 24.5 [range, 7–302] months) with chronic hemiparetic stroke who were admitted to our hospital for intensive upper extremity rehabilitation. The mean Fugl-Meyer Assessment (FMA) UE score was 28.6. All of them are independent in their activity of daily living (ADL) and without remarkable cognitive deficits. Amount-of-use score of Motor Activity Log-14 (MAL-AOU) was applied as the index of daily use of affected UE. The following parameters were examined as the explanatory variables: demographics, proximal and distal sub-scores of FMA UE, Modified Ashworth Scale (MAS), and sensory function scores in the Stroke Impairment Assessment Set (SIAS).

**Results:**

The median MAL-AOU score was 0.57 [range, 0.28–0.80]. Ordinal regression analysis revealed that FMA proximal, FMA distal, and SIAS sensory function (touch) were associated with AOU score of MAL-14 (Pseudo R-square = 0.460).

**Conclusion:**

Not only motor but also sensory function, especially tactile sensation, play a crucial role in the daily use of affected UE in chronic stroke patients with severe UE hemiparesis.

## Introduction

In chronic stroke patients, the amount of affected upper extremity (UE) use is important not only to maintain functional ability but also prevent “learned nonuse” and subsequent functional deterioration [1]. Although a growing number of reports demonstrate the efficacy of specific intensive approaches on certain aspects of disability, particularly regarding UE function, it is noteworthy that the benefit is in general limited for the patients with chronic stroke [2]. On the other hand, approaches to proceed a self-management or home-based rehabilitation has a certain effect not only to maintain chronic health condition, but also to improve body function [3,4]. It is suggested that tailored counselling or with tailored supervised training as well as nurses’ intervention improves participation and arm function [5,6].

To design a more personalized home-based rehabilitation program, it is imperative to know the factors affecting paretic UE disuse in activities of daily living (ADL) in the chronic stage. Such factors may be the treatment target of telehealth intervention [7]. Researchers have reported a predictability of daily use or activity limitation in the chronic phase using motor functional profile in the sub-acute phase [8,9], but it should be noted these studies reported indirect relationship in the different phases of stroke. Just two other studies have examined the contributions of motor function and proprioception individually in single factorial analyses [10,11]. Thus, no study has so far attempted to reveal the direct real-time causal relationship between UE use and clinically important impairment profiles, including motor function, spasticity, tactile sensation, and proprioception, with multifactorial analysis [12]. Subsequently, a responsive intervention to improve the participation of upper extremity targeting specific aspect of sequelae remains difficult.

On these grounds, the present retrospective cross-sectional study aimed to determine which clinical factors influence the paretic UE participation in chronic hemiparetic stroke patients, particularly those with severe motor and mild-to-moderate sensory disturbances.

## Material and method

### Participants

We retrospectively reviewed the medical records of 138 consecutive patients with chronic hemiparetic stroke who were admitted to Keio University Hospital for intensive UE rehabilitation studies between October 2015 and October 2018. The Ethics Review Board at Keio University School of Medicine approved this retrospective survey as well as the initial intervention studies, which were conducted in accordance with the Declaration of Helsinki. All enrolled chronic stroke patients provided a written informed consent prior to their inclusion in the study for the collection, usage of their data, and publication of their findings in this study and any further non-specified retrospective studies thereafter. In addition, a notification about the implementation of this study was posted on the bulletin board at outpatient clinic and on the homepage of the hospital website according to the national guideline.

The inclusion criteria were: (i) largely severe UE hemiparesis due to stroke; (ii) at least 6 months after stroke onset; (iii) patients in the age range of 14–80 years; (iv) independence in ADL and ambulatory with/without functional or walking aids; and (v) no history of neurorehabilitation within 6 months before admission. The exclusion criteria were: (i) history of major psychiatric or previous neurological disease; (ii) severe pain in the paretic UE; (iii) patients with implanted pacemakers or other stimulators; (iv) a Mini-Mental Scale Examination score below 25; (v) clinically apparent visuospatial neglect or apraxia; (vi) missing demographic or clinical data; and (vii) bilateral brain lesions.

It is widely recognized that the hemispatial neglect affects patients’ ADL and social participation [13] and that the more severe visuospatial neglect the patient has, the stronger interaction between motor and sensory deficit is induced [12]. Thus, we included a population without cognitive dysfunction to more clearly delineate the features of disuse and functional status according to a similar study on subacute stroke [14]. We excluded patients with a pacemaker or other implant stimulator to ensure safety during the initial examinations utilizing peripheral electrical stimulation and/or transcranial magnetic stimulation.

### Data acquisition

Data collected at admission included age, sex, side of hand dominance, impaired side of the body, and duration of stroke. Clinical data were assessed before the UE intervention. Amount-of-use score of Motor Activity Log-14 (MAL-AOU) was administered to evaluate the frequency of paretic UE use. The MAL-14 is a valid and reliable semi-structured interview that elicits information on 14 representative ADL, and the AOU score namely represents the amount of impaired arm use to accomplish ADL from various aspects. The items were scored from 0 to 5 with lower scores indicating inability to complete the ADL task with the affected UE [1]. An average score of all 14 items was calculated for the final analysis. Demographics regarding age, sex, side of hand dominance, impaired side of the body, duration of stroke, motor and sensory function, and spasticity were collected from the patients’ medical records. Motor function was assessed using the Fugl-Meyer Assessment scale (FMA)-UE motor score, which comprises 33 items with scores ranging from 0 to 66 with higher scores indicating more normal movement of the affected UE; we separated scores into proximal (items A: shoulder and elbow, and D: coordination score: 42) and distal (items B: hand, and C: wrist score: 24) portions for statistical analyses [15,16]. Sensory function (touch and proprioception) was measured using sensory scores in SIAS, in which tactile sensation and proprioception were scored from 0 to 3 [17,18]. Spasticity was scored using Modified Ashworth Scale (MAS) for the elbow, wrist, and finger joints, respectively. Spasticity was scored using the MAS [19]. MAS scores were transformed from 1+ to 2, 2 to 3, and 3 to 4, and the summation of these joints were used as the MAS UE flexor score for the analysis.

### Analysis

The amount of paretic UE use in ADL, scored with MAL-AOU, was analyzed as a dependent variable while other parameters were analyzed as explanatory variables. Descriptive statistics of the demographics, stroke data, and clinical measures were calculated. Since the MAL-AOU and several clinical measures were not normally distributed (Shapiro-Wilk test <0.05), the median and interquartile range (IQR) were calculated and nonparametric tests were used. The association between the MAL-AOU and other factors were analyzed using Spearman’s rank correlation coefficients for continuous variables. Differences in MAL-AOU between the two groups (male vs female; affected dominant vs nondominant hand) were examined using Mann-Whitney U tests for dichotomous variables.

Variables were preselected for regression analysis according to Spearman’s correlation coefficients (rho). All variables with rho values more than 0.15 were included as covariates in an ordinal logistic regression model with backward Wald selection (excluding values with *P* > 0.10) to identify factors associated with MAL-14. When highly related variables (rho > 0.5) existed, only one of them was applied for the regression analysis to avoid multicollinearity problems, while no variable met the condition in the present study [20]. All data were analyzed using IBM SPSS Statistics 25 (IBM Corp., Armonk, NY, USA). Values with *P* < 0.05 were regarded as statistically significant.

## Results

The severity of participants’ UE disability scores ranged from level 1a to 2 on the finger function scoring of SIAS. The exclusion criteria for this retrospective survey comprised of (a) missing demographic or clinical data (n = 15) and (b) bilateral brain lesions (n = 5). Data from 117 patients with chronic stroke (media age, 55 [range, 18–85] y; median stroke duration 25 [range, 6.7–302.0] months) were finally analyzed.

The patient characteristics are summarized in Table 1. The MAL-AOU score was significantly correlated with the FMA proximal and distal; SIAS touch and proprioception; and MAS scores (*P* < 0.05, Table 2). Variables having rho values more than 0.15: scores of FMA proximal and distal, SIAS light touch and proprioception, and MAS were included in the regression analysis as covariates (i.e. explanatory variable) in an ordinal regression model (excluding values with *P* > 0.10) to identify factors associated with MAL-AOU as a dependent variable. Ordinal logistic regression analysis revealed that FMA proximal (Wald = 25.15, *P* < 0.001), FMA distal (Wald = 9.86, *P* < 0.01) and SIAS touch (Wald = 8.58, *P* < 0.01) were independently correlated with the daily use of the affected UE (Pseudo R-square = 0.460; Table 3).

**Table 1:** Characteristics of patients with chronic stroke (n = 117).

	Median (IQR)
**Age (y.o.)**	55 (47–64)
**Stroke duration (mo)**	25.33 (15.43–41.70)
**Gender (n)**	M 68: F 49
**Side of lesion (n)**	Rt 55: Lt 62
**Dominant hand (n)**	Rt 110: Lt 7
**Dominant hand affected (n, %)**	66 (56.4%)
**MAL-AOU (max 5)**	0.57 (0.29–0.79)
**FMA-UE total score**	27 (20–35)
**FMA-UE proximal sub-score (max 42)**	21 (15–25)
**FMA-UE distal sub-score (max 24)**	7 (4–11)
**MAS UE flexor score (max 6)**	4 (2–6)
**SIAS-S light touch (max 3)**	2 (2–3)
**SIAS-S position (max 3)**	3 (1–3)

The patient characteristics are shown. Chronic stroke patients with severe upper extremity paresis are included in this study.

Abbreviations: IQR, interquartile range; MAL-AOU, Amount-of -use score of Motor activity log-14; FMA, Fugl-Meyer Assessment scale; UE, Upper Extremity; MAS, modified Ashworth scale; SIAS-S, sensory items of Stroke impairment assessment set.

**Table 2:** Spearman’s rank correlation coefficients between MAL-AOU and other factors for continuous variables.

	rho	*P*
Age (months)	0.114	*0*.*220*
Stroke duration (months)	-0.117	*0*.*208*
FMA Proximal score	0.568^†^	*<0*.*001*
FMA Distal score	0.495^†^	*<0*.*001*
MAS UE flexor score	-0.263^†^	*0*.*005*
SIAS-S (UE) Light touch score (max 3)	0.320^†^	*<0*.*001*
SIAS-S (UE) Proprioception score (max 3)	0.184*	*0*.*047*

The MAL-AOU score was significantly correlated with the FMA proximal and distal; SIAS touch and proprioception; and MAS scores.

Abbreviations: MAL-AOU, Amount-of -use score of Motor activity log-14; FMA, Fugl-Meyer Assessment scale; UE, Upper Extremity; MAS, modified Ashworth scale; SIAS, sensory items of Stroke impairment assessment set.

**Table 3:** Ordinal logistic regression analysis of factors associated with MAL-AOU.

	Wald	*P*
FMA proximal score	25.333	<0.001
FMA distal score	9.023	0.003
SIAS light touch (UE)	9.046	0.003
SIAS proprioception (UE)	0.754	0.385
MAS	0.189	0.664

A logistic regression analysis revealed that FMA proximal, FMA distal and SIAS touch were independently correlated with the daily use of the affected UE.

Abbreviations: MAL-AOU, Amount-of -use score of Motor activity log-14; FMA, Fugl-Meyer Assessment scale; UE, Upper Extremity; SIAS, Stroke impairment assessment set; MAS, modified Ashworth scale.

## Discussion

The present study demonstrated that both motor and tactile sensory functions were independently associated with the amount of daily use of the affected UE in patients with chronic hemiparetic stroke. The UE paralysis was severe among the studied population (mean FMA score: 28.6) with low participation in their ADL (mean MAL-AOU value: 0.595). Thus, the participants with chronic stroke sequelae rely almost only on their non-paretic UE to perform ADL. These results are consistent with previous reports regarding motor paralysis [10,21], and sensory disturbance in the subacute phase [22]. Because a somatosensory loss makes stroke survivors unable to differentiate limb positions or the shape, size, hardness, texture, or weight of objects [22,23], this loss typically negatively impacts their ability to grasp and manipulate objects [24]. Kong et al. identified UE dexterity in 28.3% of a cohort of patients with chronic stroke and found that sensory impairment significantly correlated with poor UE dexterity [25]. Turville et al. indicated that changes in functional arm use after retraining for stroke-related somatosensory loss were associated with some variance in somatosensory outcomes [14]. In addition, the present study newly revealed that the existent tactile sensation is one of the major determinants of ADL participation of the affected UE.

The fact that not only motor but also tactile sensation affects the daily use of paretic extremity emphasize the importance of training strategy targeting tactile sensation in the chronic stroke patients. Tactile sensory function as represented by discriminative touch is associated with dexterity in mild-to-moderate stroke patients in subacute-to-chronic phase [26]. However, it may be also true that the sensory recovery has been frequently overlooked in the clinics [27,28]. While a number of researchers reported the effect of sensory trainings, it is still controversial to which degree the training resources and time should be divided onto the sensory matter with sacrificing the motor trainings. Therefore, as precision medicine widely attracts scientific and clinical attention these days, we may need to establish precision rehabilitation, a scientific evidence-based more personalized rehabilitation over the patient’s personal background factors which includes not only biological aspects but also behavioral characteristics, to find the best answer for each individual patients [29]. Further researches are needed to resolve the trade-off relation between sensory and motor training, and to propose the best rehabilitative prescription composed of best mixture of these trainings for each patient.

In order to proceed both of sensory training and daily usage of paretic upper extremity, we consider home-based rehabilitation could be a good measure without affecting the resources for motor-targeting rehabilitation. While there are a number of reports on the sensory-targeted rehabilitation in chronic stroke patients, the majority of these interventions were done on the hospital/center basis [27,28]. However, despite only very few researches reported, it might be originally suited for home-based and/or self-training because sensory training originally needs less physical assistance [30,31]. Sumania, et al., have treated chronic patients at home with traditional passive sensory tasks composed of tactile discrimination of materials, recognition of objects and weight, and proprioception trainings of joint position, fine and gross movements [30]. Sullivan and Hedman reported a sensorimotor training including sensory amplitude stimulation to apply electrical stimulation below muscle contraction level [31]. These approaches seems suited on this purpose. Studies are needed to reveal the effect of home-based sensory training on the aspect of daily usage of paretic limb.

It is unclear why the superficial rather than the positional sense was associated with the participation of the paralyzed UE in this study. Our findings were similar to those of previous studies [14] in which changes in arm use after somatosensory retraining were associated more with tactile sensation than proprioception. Conversely, a systematic review showed that proprioception significantly correlated with perceived level of physical activity and social isolation [32]. In addition, a recent retrospective study has reported that proprioception is strongly correlated with MAL score as well as various motor functional parameters [11]. We might consider that a strong correlation between motor function and proprioception may be one of the reasons why the involvement of position sensation was not detected on the regression analysis in our study. In addition, the present results may imply that the involvement of each sensory modality in disuse can differ according to the phenotype and/or severity of impairments.

The present study has some potential limitations. First, since the MAS-AOU is a self-report measurement, there could be recall and response bias as well as the capacity to over- or underestimate true physical activity [33,34]. Second, our study was conducted at a single institution. Third, the findings will not be generalizable to the general population with stroke due to the severe UE paresis in the patients of our study. Fourth, sensory disorders were assessed not with a quantitative but with a semi-quantitative method. Recently, as the progress of technological innovation, a number of researchers are utilizing the wearable sensors/accelerometers [35–37]. Therefore, a larger prospective cohort study that can ensure a subgroup analysis of various impairment profiles, with detailed and quantitative assessment of sensory disturbances utilizing will be needed.

## Conclusions

In conclusion, both motor and sensory, especially tactile, functions play a crucial role in the daily use of affected UE in patients with chronic stroke. Our results will emphasize the importance of sensory training especially at the home-basis among chronic stroke patients and might provide an important insight for developing precision rehabilitation.

## Supporting information

S1 File (XLSX)

## Abbreviations

UE: upper extremity
ADL: activities of daily living
MAL-AOU: Amount-of-use score of Motor activity log-14
SIAS: Stroke impairment assessment sets
FMA: Fugl-Meyer assessment scale
MAS: modified Ashworth scale
